# The characteristics of latent tuberculosis infection and its influencing factors in hospitalized patients in Suzhou, Jiangsu, China

**DOI:** 10.1371/journal.pone.0322913

**Published:** 2025-06-17

**Authors:** Yanyang Zhou, Xing Lv, Shuai Zhu, Ping Xu

**Affiliations:** 1 Suzhou Medical College of Soochow University, Suzhou, Jiangsu, China; 2 Department of Clinical Laboratory, The Fifth People’s Hospital of Suzhou, Suzhou, Jiangsu, China; Shandong Public Health Clinical Center: Shandong Provincial Chest Hospital, CHINA

## Abstract

**Objective:**

China is a country with a high burden of tuberculosis (TB). It is vital to reduce the number of new cases of TB in China. We aimed to examine and investigate the distribution and affecting factors of the latent tuberculosis infection (LTBI) detection rate in hospitalized patients in Suzhou, Jiangsu Province.

**Methods:**

We analyzed the link between LTBI and patients’ information, disease diagnosis, and blood routine indices of hospitalized patients at the Fifth People’s Hospital in Suzhou from January 1, 2018, to March 31, 2024.

**Results:**

Results indicated that of the 6692 patients included in the study, 39.05% of them were diagnosed with LTBI. Multivariate analysis revealed that sex, AIDS status, testing time, age, lymphocyte count, and neutrophil count were influencing factors for the detection of LTBI ( p < 0.05). However, hepatitis B, diabetes, hypertension, silicosis and monocyte count did not significantly influence LTBI detection.

**Conclusion:**

Sex, AIDS status, testing time, age, lymphocyte count, and neutrophil count were influencing factors for the detection of LTBI.

## Introduction

Tuberculosis (TB) is an infectious disease caused by *Mycobacterium tuberculosis* (Mtb) that most often affects the lungs. It spreads through the air when people with TB cough, sneeze or spit [1]. According to the World Health Organization (WHO) ‘s Global Tuberculosis Report 2024, TB has become the world’s leading cause of infectious disease related death, with more than 10 million new cases reported each year [1]. TB is classified into two types: active TB (ATB) and inactive TB or latent TB infection (LTBI) [2]. China has a double-high burden of ATB and LTBI cases. In 2023, approximately 741,000 new TB cases were reported in China, representing 6.86% of the global total and placing the country third worldwide [1]. The Chinese Center for Disease Control report highlights that the LTBI rate among individuals aged 15 and older stands at around 20.30% [3]. LTBI refers to a state of persistent immune response to Mtb specific antigen without clinical TB evidence [4]. It is estimated that about a quarter of the global population has been infected with TB, and 5% to 10% of LTBI will gradually develop into ATB [5,6]. Previous studies [7–9] have shown that immunosuppression, anemia, comorbidities and high IGRA index levels are risk factors for ATB progression to LTBI. The LTBI population is at high risk for developing new-onset ATB. Consequently, early identification and interventions in the LTBI population are crucial for reducing TB incidence and achieving the WHO end TB target [6].

Common laboratory diagnostic methods for LTBI include interferon gamma release assay (IGRA) test, and tuberculin skin test (TST) [10]. The sensitivity and specificity of IGRA are superior to those of TST, and IGRA provides results based on in vitro interpretation. Overall, IGRA is more effective than TST in diagnosing LTBI [11–13]. IGRA is a cellular immune-based method to detect interferon-γ (IFN-γ) released by peripheral blood cells under the stimulation of Mtb antigen. The concentration of IFN-γ determines whether a peripheral blood donor has specific cellular immunity for Mtb, indicating TB infection [14]. IGRA testing and preventive treatment in high-risk populations is an effective strategy for TB control [15,16]. Therefore, it is of great significance to clarify the high-risk population of IGRA positive results and explore the factors affecting IGRA test results for the control and management of LTBI populations, as well as the correct interpretation of LTBI diagnosis results.

Previous studies have demonstrated that advanced age [17], smoking [18], diabetes [19,20], hypertension [21,22] and obesity [23] are significant risk factors for LTBI. However, these studies were limited in scope regarding the factors considered and the sample sizes utilized. This study expands upon prior research by incorporating detection time and peripheral blood cell counts as additional variables, thereby enriching content of previous studies. This paper examined the detection rate of LTBI in hospitalized patients at Suzhou Fifth People’s Hospital from January 1, 2018, to March 31, 2024, along with the factors that influenced it. It aimed to explore the factors affecting the IGRA test results such as patients’ conditions and clinical indicators in Suzhou city, in order to enhance the interpretation of IGRA results and improve the diagnosis of LTBI in clinical practice.

## 1. Methods

### 1.1 Study population

**Fig 1:**
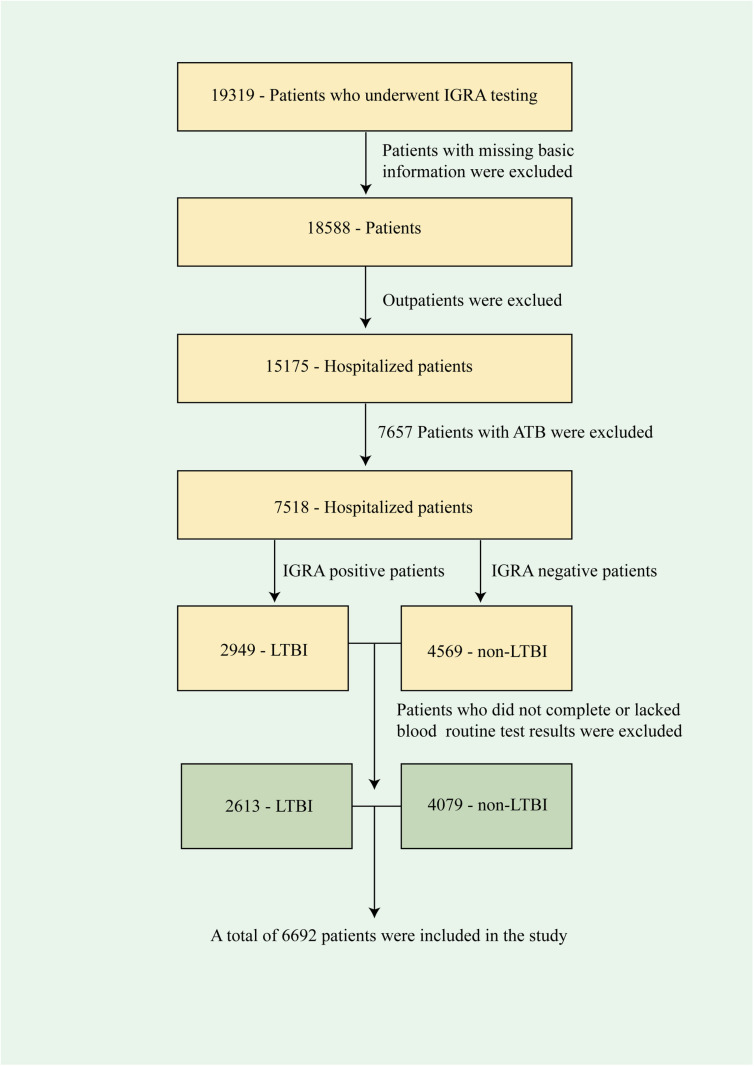
Flow diagram of the study population.

This study was conducted at Suzhou Fifth People’s Hospital, in the Suzhou Clinical Medical Center for Infectious Diseases. Basic information such as gender and age of hospitalized patients receiving IGRA from January 1, 2018, to March 31, 2024, was collected, as well as the diagnosis of TB, acquired immune deficiency syndrome (AIDS), diabetes, hepatitis B, hypertension, silicosis, and other diseases. Patients with missing data on their basic information were excluded. Patients diagnosed with ATB were also excluded. In addition, to investigate the impact of peripheral blood cell types on LTBI, blood routine test results from patients who underwent IGRA were collected within the same time frame (within 7 days). Patients who did not undergo blood tests during the same period were excluded, leaving 6692 patients (Fig 1).

### 1.2 Diagnostic criteria

Diagnosis of the disease was performed by the clinician. The diagnostic criteria included in the analysis were as follows: (1) ATB: According to the Chinese health industry standard WS288–2017 “Diagnosis of TB”, the patient exhibited clinical symptoms and signs consistent with TB, and was diagnosed with ATB based on etiological evidence of Mtb, pathological findings, and imaging studies. (2) LTBI: according to Chinese health industry standard WS288–2017 “Diagnosis of TB”. A positive IGRA and exclusion of current TB disease and previous history were considered to indicate LTBI. (3) AIDS: according to Chinese health industry standard WS293–2008 “Diagnostic Standards for AIDS”. Human Immunodeficiency virus (HIV) antibody confirmed positive or HIV strain isolated from blood; history of acute HIV epidemic syndrome or epidemiology, and two positive HIV nucleic acid tests at different times. Individuals who meet either of the aforementioned criteria, along with a CD4^+^ T lymphocyte count below 200 cells/μL and the presence of an AIDS-defining illness, are diagnosed with AIDS. (4) Hepatitis B infection: according to Chinese health industry standard WS299–2008 “Diagnostic Standards for Viral Hepatitis B”. Patients with HBV serology and DNA diagnosis are considered hepatitis B infection. (5) Diabetes mellitus: Based on China’s Type 2 Diabetes Prevention and Treatment Guidelines. Fasting blood glucose of 7.0 mmol/L, blood glucose of 11.1 mmol/L after 2 hours of glucose load, random blood glucose of 11.1 mmol/L, or glycated hemoglobin of 6.5% or more. Those who meet one of the conditions listed above and have typical diabetic symptoms are considered diabetes mellitus. (6) Hypertension: According to the Chinese Guidelines for the Prevention and Treatment of Hypertension, patients with systolic blood pressure of 140 mm Hg and/or diastolic blood pressure of 90 mmHg are considered hypertensive. (7) Silicosis: according to Chinese health industry standard GBZ70–2015 “diagnosis of occupational pneumoconiosis”. Based on the reliable and productive history of mineral dust contact after X-ray photography of the chest, combined with workplace occupational hygiene, pneumoconiosis epidemiology investigations, and occupational health monitoring data, reference to clinical manifestations and laboratory examinations, and excluding other similar lung diseases, the control standards for pneumoconiosis diagnosis can be used to diagnose patients with silicosis.

### 1.3 IGRA detection and result interpretation

Based on the QuantiFERON-TB Gold In-Tube principle, the IGRA test kit (provided by Wantai, China) utilized at the Fifth People’s Hospital of Suzhou was employed to interpret the IGRA results based on the values of the N, P, and T tubes. The criteria for determining the IGRA results are as follows: (1) For a positive result: N tube ≤400 pg/ml; T-N ≥ 14 pg/ml; and T-N ≥ N/4. (2) For a negative result: N tube ≤400 pg/ml; P-N ≥ 20 pg/ml; T-N ≥ 14 pg/ml and T-N < N/4. (3) For a negative result: N tube ≤400 pg/ml; P-N ≥ 20 pg/ml; T-N < 14 pg/ml. (4) All other scenarios are classified as indeterminate.

### 1.4 Data collection

Patient data were obtained from the information management system and inspection information management system of our hospital. The data access remains valid on a long-term basis starting from May 10, 2023. Information on inpatients receiving IGRA from January 1, 2018, to March 31, 2024. One person with expertise in data collection and processing performed the initial collection and data summary, while another person checked, screened, and statistically converted the aggregated data.

### 1.5 Statistical analysis

SAS 9.4 was used for statistical analysis, and Prism 9.0 was used for mapping. Categorical data were expressed as percentage (%) and measurement data as median (25% and 75% quartiles). The Mann-Whitney was used to compare the continuous variables by nonparametric test; the chi-square test to compare the rates of univariate classification variables; Pairwise comparisons of rates for univariate categorical variables were performed by Bonferroni correction. Binary logistic regression was performed using data of 6692 patients’ results for LTBI diagnosis as the dependent variable and patient characteristics as independent variables, deriving odds ratios (ORs) values and 95% confidence intervals (95% CIs). Statistical analyses were two-sided, with P < 0.05 indicating statistical significance except for pairwise comparisons.

### 1.6 Ethics approval

The research, related to human use, complied with all the relevant national regulations, and institutional policies and in accordance the tenets of the Helsinki Declaration, and has been approved by the Ethics Committee of the Fifth People’s Hospital of Suzhou (2023) Hospital ethics Review No. (012). The ethics committee waived the need for informed consent. Data access was effective on May 10, 2023 (long-term validity). Data collection for the study was completed for the retrospective analysis in April 2024.

## 2. Results

### 2.1 Study population

Of the 6692 patients who received IGRA testing, 2,613 were diagnosed with LTBI, a detection rate of 39.05%. The LTBI population included 2,114 (80.90%) men and 499 (19.10%) women. Of the LTBI population, 380 (14.54%) had AIDS, 110 (4.21%) had hepatitis B, 346 (13.24%) had diabetes, 985 (37.70%) had hypertension, and 998 (38.19%) had silicosis (Table 1).

**Table 1:** Basic population characteristics of the 6692 patients included in the study.

Factors	Groups	LTBI populations (proportion)	Non-LTBI populations (proportion)	Detection rate of LTBI
		2613 (39.05%)	4079 (60.95%)	
Sex	Man	2114 (80.90%)	3196 (78.35%)	39.81%
	Woman	499 (19.10%)	883 (21.65%)	36.11%
Age (year)	<25	178 (6.81%)	150 (3.68%)	54.27%
	25 ~ 44	571 (21.85%)	924 (22.65%)	38.19%
	45 ~ 64	796 (30.46%)	1337 (32.78%)	37.32%
	>64	1068 (40.87%)	1668 (40.70%)	39.04%
AIDS	Yes	380 (14.54%)	965 (23.66%)	28.25%
	No	2233 (85.46%)	3114 (76.34%)	41.76%
Hepatitis B	Yes	110 (4.21%)	145 (3.55%)	43.14%
	No	2503 (95.79%)	3934 (96.45%)	38.88%
Diabetes mellitus	Yes	346 (13.24%)	525 (12.87%)	39.72%
	No	2267 (86.76%)	3554 (87.13%)	38.95%
Hypertension	Yes	985 (37.70%)	1309 (32.09%)	42.94%
	No	1628 (62.30%)	2770 (67.91%)	37.02%
Silicosis	Yes	998 (38.19%)	1200 (29.42%)	45.40%
	No	1615 (61.81%)	2879 (70.58%)	35.94%
Period (Year-month-day)	2018-1-1 to 2019-12-31	1032 (39.49%)	1053 (25.82%)	49.50%
	2020-1-1–2023-3-31	1305 (49.94%)	1194 (29.27%)	52.22%
	2023-4-1–2024-3-31	276 (10.56%)	1832 (44.91%)	13.09%

Abbreviations: LTBI: latent tuberculosis infection; AIDS: acquired immune deficiency syndrome.

### 2.2 Blood cell count characteristics of the study population

**Fig 2:**
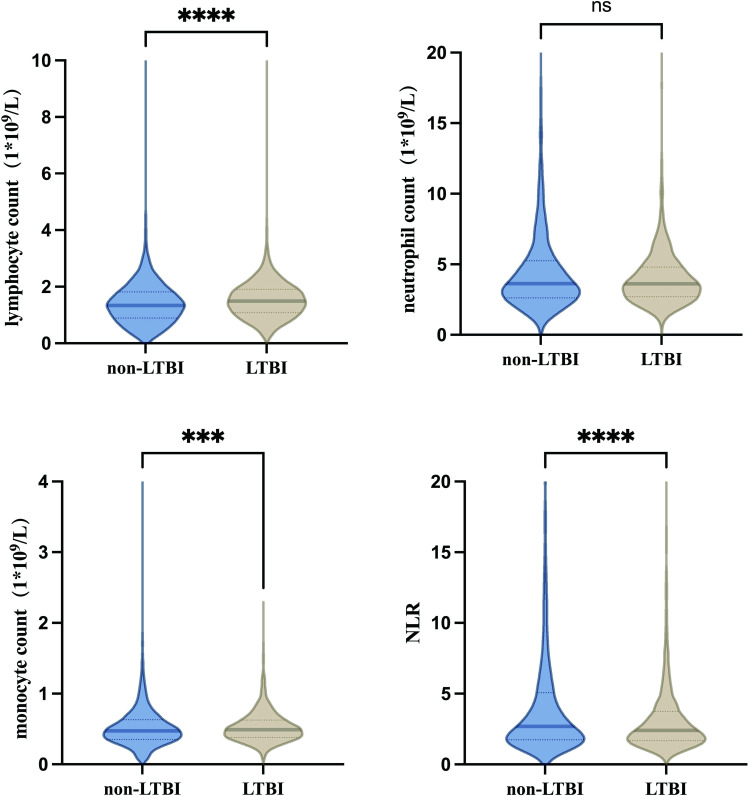
Distribution of blood routine indexes in LTBI and non-LTBI populations.

Blood count analysis revealed that the lymphocyte count in the LTBI group (1.340 [0.890, 1.820]) was significantly lower compared to the non-LTBI group (1.490 [1.080, 1.910]) (p < 0.0001). Conversely, the monocyte count in the LTBI group (0.472 [0.351, 0.630]) was significantly higher than in the non-LTBI group (0.491 [0.381, 0.624]) (p < 0.001). There was no significant difference in neutrophil counts between individuals with LTBI (3.629 [2.618, 5.256]) and those without LTBI (3.614 [2.709, 4.799]) (p = 0.687) (Table 2) (Fig 2).

**Table 2:** Cell count characteristics of different populations.

Group	Lymphocyte count (1 * 10^9^/L)	Neutrophil count (1 * 10^9^/L)	Monocyte count (1 * 10^9^/L)	NLR
LTBI populations	1.340 (0.890, 1.820)	3.629 (2.618, 5.256)	0.472 (0.351, 0.630)	2.692 (1.742, 5.071)
Non-LTBI populations	1.490 (1.080, 1.910)	3.614 (2.709, 4.799)	0.491 (0.381, 0.624)	2.402 (1.684, 3.753)
p value	<0.0001	0.069	<0.001	<0.0001

Abbreviations: NLR (Neutrophil to lymphocyte ratio). Cell counts and the NLR were reported as medians with interquartile ranges (IQR). The Mann-Whitney U test was employed to assess differences in cell counts and NLR between the LTBI and non-LTBI populations.

### 2.3 The effect of different age and gender groups on the detection rate of LTBI

**Fig 3:**
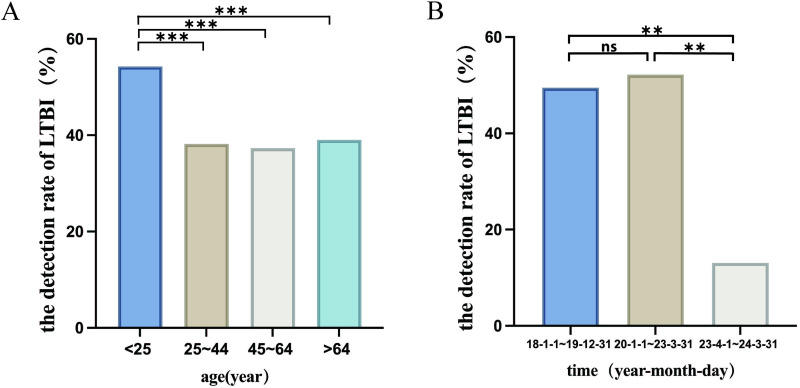
Differences in LTBI detection rates among different groups. **A.** Differences in the detection rate of LTBI in patients of different age groups. **B.** Differences in the detection rate of LTBI in patients at different testing periods.

**Fig 4:**
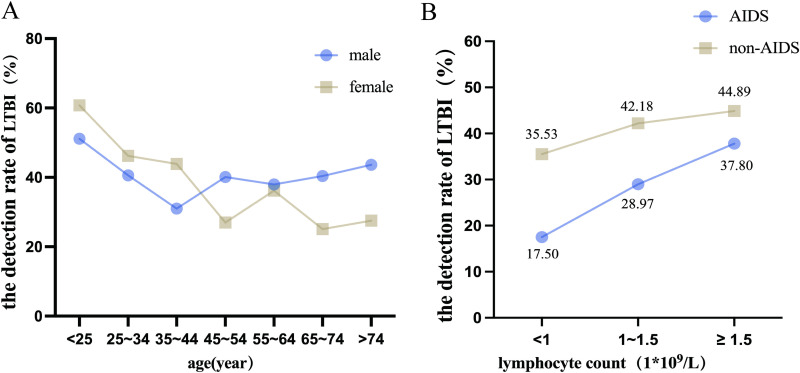
Distribution of LTBI detection rates in different groups. **A.** Distribution of LTBI detection rates among patients of different ages and genders. **B.** Distribution of LTBI detection rate between AIDS patients and non-AIDS patients with different lymphocyte counts.

The detection rate of LTBI was significantly higher in men than in women (*X*^2^ = 6.323, p = 0.012). The detection rate of LTBI was compared across age groups by Bonferroni correction. The result showed that individuals under the age of 25 had a significantly higher LTBI detection rate (54.27%) compared to other age groups (p < 0.0001) (Fig 3A) (Table 3), and there was no significant difference in the detection rate of LTBI across other age intervals. Based on the distribution of LTBI detection rates across different genders and age groups, we observed that the detection rate of LTBI in female patients consistently decreased with advancing age. In contrast, male patients exhibited a higher detection rate in both younger and elderly age groups, while showing a lower detection rate in the middle-aged group (Fig 4A).

**Table 3:** Group comparison of LTBI detection rate among different age groups.

Comparison group 1		Comparison group 2		*X* ^*2*^	p value
Age (year)	LTBI rate	Age (year)	LTBI rate		
<25	54.27%	25 ~ 44	38.19%	28.713	< 0.0001
		45 ~ 64	37.32%	34.154	< 0.0001
		>64	39.04%	28.168	< 0.0001
25 ~ 44	38.19%	45 ~ 64	37.32%	0.287	0.308
		>64	39.04%	0.288	0.307
45 ~ 64	37.32%	>64	39.04%	1.495	0.116

Bonferroni correction was used to compare the difference in LTBI detection rate among different age groups.

### 2.4 Effect of different diseases on the positive detection rate of LTBI

Univariate analysis of the difference in the detection rate of LTBI between a total of 6692 patients showed that LTBI was related to the presence of AIDS, hypertension, and silicosis (p < 0.05). The patients’ AIDS status had a significant effect on the positive detection rate of LTBI. The positive detection rate of LTBI in AIDS patients was 28.25%, significantly lower than the detection rate of 41.76% in the uninfected population (p < 0.001). The presence of hypertension is also a factor that influences the detection rate of LTBI. The detection rate of LTBI in hypertensive patients was 42.94%, significantly higher than the detection rate of 37.02% in people without hypertension (p < 0.001). Additionally, the detection rate of LTBI in the population with silicosis was 45.40%, while the detection rate of LTBI in the population without silicosis was 35.94%. The difference was statistically significant (p < 0.001) (Table 4).

**Table 4:** Table presenting a univariate analysis of the impact of various diseases on the detection rate of LTBI.

Factors	Groups	LTBI populations	Total number of cases	LTBI rate	*X* ^*2*^	p value
Hepatitis B infection	Yes	110	255	43.14%	1.864	0.172
	No	2503	6437	38.88%		
AIDS	Yes	380	1345	28.25%	82.402	0.001
	No	2233	5347	41.76%		
Diabetes mellitus	Yes	397	922	43.06%	0.502	0.479
	No	2555	6109	41.82%		
Hypertension	Yes	985	2294	42.94%	22.210	<0.001
	No	1628	4398	37.02%		
Silicosis	Yes	998	2198	45.40%	55.597	<0.001
	No	1615	4494	35.94%		

Chi-square test was used to compare the influence of various disease factors on the detection rate of LTBI.

Fig 4B shows the AIDS status and lymphocyte count distribution of the study population. It turned out that the detection rate of LTBI in AIDS patients was significantly lower than that of non-AIDS populations. As lymphocyte count increased, both patients with AIDS and non-AIDS populations showed an increase in the detection rate of LTBI.

### 2.5 Effect testing period on the detection rate of LTBI

According to the changes in management policy of hospitalized patients and the changes in people’s health habits over time, the IGRA testing period was divided into three sections, that is, January 1, 2018, to December 31, 2019, January 1 2020, to March 31, 2023, and April 1, 2023, to March 31, 2024. The detection rates of LTBI in the three periods were 49.5%, 52.22%, and 13.09%, respectively. Pairwise comparison of the detection rate of LTBI in different periods after adjusting the α’ significance level. The results showed that the detection rate of LTBI from 01 January 2018 to 31 December 2019 and 01 January 2020 to 31 March 2023 were significantly higher than those from 01 April 2023 to 31 March 2024 (P < 0.0167). However, there was no significant difference in the detection rate of LTBI from 01 January 2018 to 31 December 2019 and 01 January 2020 to 31 March 2023 (p = 0.066) (Fig 3B).

### 2.6 Multivariate analysis of determinants of LTBI detection

The outcomes of patients’ LTBI diagnosis were considered as the dependent variable Patients diagnosed with LTBI were assigned a value of 1, while those without LTBI were assigned a value of 0. First, we calculated the unadjusted effect of each independent variable on the likelihood of a patient being diagnosed with LTBI to obtain crude ORs. Subsequently, all variables were incorporated into a multivariate analysis to control for potential confounding factors, yielding adjusted ORs (AORs) (Table 5).

**Table 5:** Table of the multivariate logistic regression results.

Variable	Factor	Reference	p value^a^	Crude ORs (95% CI)^a^	p value^b^	Adjusted ORs (95% CI) ^b^
Sex	Male = 2	Female = 1	0.0120	1.170 (1.035, 1.313)	<0.0001	1.332 (1.166, 1.522)
AIDS	Yes = 1	No = 0	<0.0001	0.549 (0.481, 0.645)	<0.0001	0.538 (0.464, 0.625)
Hepatitis B infection	Yes = 1	No = 0	0.190	1.184 (0.920, 1.524)	0.798	1.035 (0.794, 1.350)
Diabetes mellitus	Yes = 1	No = 0	0.681	1.031 (0.891, 1.193)	0.473	0.946 (0.809, 1.106)
Hypertension	Yes = 1	No = 0	<.0001	1.279 (1.154, 1.417)	0.084	1.112 (0.986, 1.254)
Silicosis	Yes = 1	No = 0	<.0001	1.481 (1.335, 1.643)	0.166	1.093 (0.965, 1.239)
Period (Year-month-day)	“2018-1-1 to 2019-12-31” = 3	“2023-4-1 to 2024-3-31” = 1	<.0001	2.149 (1.844, 2.504)	<0.0001	1.931 (1.650,2.260)
	“2020-1-1 to 2023-3-31” = 2	“2023-4-1 to 2024-3-31” = 1	<.0001	4.170 (3.575, 4.864)	<0.0001	4.016 (3.429,4.704)
Age (year)	Numerical variable		0.021	0.997 (0.994, 1.000)	0.015	0.996 (0.993,0.999)
Monocyte count (1 * 10^9^/L)	Numerical variable		0.347	0.954 (0.865, 1.052)	0.896	0.996 (0.917,1.105)
Lymphocyte count (1 * 10^9^/L)	Numerical variable		0.001	1.112 (1.045, 1.184)	0.012	1.091 (1.020,1.168)
Neutrophil count (1 * 10^9^/L)	Numerical variable		0.918	1.003 (0.956, 1.052)	<0.0001	0.928 (0.908,0.949)

The outcomes of patients’ LTBI diagnosis were considered as the dependent variable Patients diagnosed with LTBI were assigned a value of 1, while those without LTBI were assigned a value of 0. Binary logistic regression was performed using gender, age, AIDS, hepatitis B infection, diabetes, hypertension, silicosis, testing period, neutrophil count, monocyte count, and lymphocyte count as independent variables.

^a^ p values, ORs, and 95% CI obtained when only single variables were included in a regression model.

^b^ p values, ORs, and 95% CI obtained when all variables were included in the regression model.

The analysis results showed that the overall regression model was statistically significant (Wald *X*^2^ = 502.549, p < 0.001). Gender (AOR = 1.332, CI 1.166, 1.522), AIDS status (OR=0.538, CI 0.464, 0.625), age (OR=0.996, CI 0.993, 0.999), lymphocyte count (OR=1.091, CI 1.020,1.168), neutrophil count (OR=0.928, CI 0.908, 0.949), testing time January 1, 2018 to December 31, 2019 (OR=1.931, CI 1.650, 2.260) and January 1, 2020 to March 31, 2023 (OR=4.016, CI 3.429, 4.704) were all statistically significant influence factors for the detection of LTBI. Among men, the risk of LTBI was 1.332 times that of women, and the risk of LTBI in AIDS was 0.538 times that of non-infected persons. The risk of LTBI from January 1, 2018, to December 31, 2019, and January 1, 2020, to March 31, 2023, was 1.931 and 4.016 times that from April 1, 2023 to March 31, 2024, respectively. In addition, the risk of LTBI decreased by 0.4% per age. However, for every 1*10^9^/L increase in peripheral blood lymphocyte count and neutrophil count, the risk of LTBI increased by 10.5% and decreased by 7.4%, respectively.

## 3. Discussion

This study showed that the overall detection rate of LTBI was 39.05% among hospitalized patients between 2018 and 2024. Monocyte and lymphocyte count distributions differed significantly between LTBI populations and non-LTBI populations. The detection rates of LTBI were highest and significant among those under 25 years old. Multivariate analysis revealed that male gender, non-AIDS status, testing period (January 1, 2018, to Dec 31, 2019, and January 1, 2020, to March 31, 2023), young age, high lymphocyte count and low neutrophil count were influencing factors for increased detection of LTBI. The possible reasons for the significant difference in the detection rate of LTBI caused by different factors can be summarized as follows: first, the factors make the population susceptible to Mtb, and the high-risk population should be protected; second, the factor affects the test results of IGRA, resulting in false positives or false negatives, to accurately interpret the report, clinicians should consider the impact of such factors on IGRA outcomes.

Our analysis suggests that male gender is an influencing factor for the increased detection rate of LTBI, which may be related to the higher smoking rate among men. Smoking leads to poor immune status in the lungs and boosts exposure to Mtb [24]*.* Exposure to secondary smoke has also been associated with a higher risk of LTBI and a dose-dependent risk of ATB [25]*.* Previous studies [26] have suggested that cardiovascular diseases including hypertension [21,22] is a risk factor for an increased detection rate of LTBI, but the biological plausibility of this association remains unclear. Our study did not observe this association. It has also been shown that prolonged silica exposure causes patient susceptibility to multiple pathogens such as Mtb [27,28]. Furthermore, several studies have shown that silicosis [29,30] is one of the risk factors for the increased detection rate of LTBI in certain areas of the population, which implies that silicosis groups have a greater likelihood of developing ATB. We did not find an association between diabetes and LTBI in the present study, consistent with the findings of Chen et al. [31], but there are also studies showing a higher prevalence of diabetes in the LTBI group [19].

In this study, the detection rate of LTBI in the AIDS population was 28.25%. This is roughly consistent with the results of Zhang et al. [32]; the detection rate of LTBI in the population of HIV-Infected patients in Jiangsu Province was 26.20%. AIDS is an important factor leading to a reduced detection rate of LTBI in hospitalized patients. AIDS is an immunodeficiency syndrome resulting from the progression of HIV infection to its terminal stage [33]. The predominant strain, HIV-1, primarily targets monocytes, macrophages, CD4^+^ T cells, and antigen-presenting cells. The high affinity between CD4 molecules and HIV gp120 facilitates the fusion of the viral lipid envelope with host cell membranes and subsequent binding to the CCR5 receptor on lymphocyte surfaces [34]. AIDS causes reduce count and immune dysfunction of T lymphocytes in patients [35], While lymphocytes are the main cells producing IFN- γ in IGRA. Petruccioli et al [36] demonstrated that the depletion of CD4^+^ T cells results in a significant reduction in IFN-γ release from cellular IGRAs in individuals co-infected with TB and HIV. In addition, chronic HIV infection leads to T cell exhaustion, and the exhausted T cells exhibit a decreased ability to secrete IFN-γ [37]. In conclusion, the number and functionality of T cells in AIDS patients are significantly reduced compared to those in other patient populations, which consequently impacts the results of IGRAs.

The detection rate of LTBI did not differ significantly between the periods of loose hospital management (January 1, 2018, to December 31, 2019) and strict management (January 1, 2020, to March 31, 2023). However, the detection rate of LTBI was significantly higher from January 1, 2018, to December 31, 2019, and from January 1, 2020, to March 31, 2023, compared with April 1, 2023, to March 31, 2024. We consider that the Chinese government’s “normalized prevention and management measures of COVID-19” until December 2022 resulted in a low number of cases of COVID-19 in China until December 2022. But after the cancellation of “normalized prevention and management measures of COVID-19” in December 2022, COVID-19 spread nationwide. COVID-19 is an acute respiratory infectious disease caused by the coronavirus-2 (SARS-CoV-2) and characterized by fever, cough, and shortness of breath [38]. According to reports, COVID-19 can cause dysfunction of the immune system [39]. T cell reduction and abnormal function have been observed in COVID-19 patients [40], as well as dendritic cell antigen presentation function impairment [41] and monocyte pro-inflammatory response reduction [42]. On the other hand, due to the change in people’s hygiene habits, people are more accustomed to wearing masks in public places from the outbreak of the COVID-19 pandemic to 2023, which affects the incidence of many airborne infectious diseases [43]. This may also partly explain the decline in the detection rate of LTBI after the COVID-19 outbreak. Based on this, COVID-19 may lead to impaired immune function, which may result in false negative IGRA results. Therefore, clinicians should be more cautious in interpreting IGRA results.

Neutrophils were shown to inhibit the secretion of IFN-γ by T cells [44]*.* In the multivariate analysis observed in our study, increased neutrophil count was a risk factor for the decreased detection rate of LTBI. This could be the high neutrophil count of the patient’s peripheral blood causing a false negative IGRA result. NLR is the ratio of absolute neutrophil and lymphocyte counts in peripheral blood. Absolute neutrophil counts reflect the inflammatory state of the body, and absolute lymphocyte counts reflect the immune regulation pathway of the body. Therefore, NLR can better represent the human’s inflammatory state [45]. In our study, the distribution of NLR in the LTBI populations and non-LTBI populations was significantly different, which was consistent with the study results of Wang et al. [46]. In addition, Madan et al. [47] found that the neutrophil counts were significantly lower in the LTBI populations than in the non-LTBI populations and that the lymphocyte counts were vice versa. It suggests that NLR may serve as a potential indicator of LTBI to reflect the inflammatory status of latent infection with TB in patients. The role of neutrophils and lymphocytes in TB needs further investigation.

The LTBI population is a large “supplementary population” of ATB patients, and early detection and intervention with the LTBI population is critical for TB prevention and control. IGRA is a common laboratory diagnostic method for the LTBI population. The specific mechanism of how LTBI may progress to ATB is currently unclear [48], But due to the huge population base of LTBI, it is important for the early diagnosis and preventive treatment of LTBI. Preventive treatment for the LTBI population can significantly reduce the incidence of ATB [15,16]. Thus, it is vital to pay close attention to the high-risk groups of IGRA positives and evaluate the factors influencing IGRA detection. It has significant implications for tuberculosis prevention and treatment.

## 5. Conclusion

In conclusion, our paper revealed that male sex, non-AIDS status, testing time (January 1, 2018, to December 31, 2019, and January 1, 2020, to March 31, 2023), low age, high lymphocyte count, and low neutrophil count were risk variables for the high detection rate of LTBI. Given the influencing factors above, the high-risk groups of LTBI, as well as the factors influencing IGRA test results, should be prioritized in TB prevention and treatment efforts.

## Supporting information

S1 File The original data of the population included in the study. (XLSX)
